# Emerging NPR-B agonists for cartilage and bone: from CNP biology to translational compound design

**DOI:** 10.3389/fchem.2026.1949456

**Published:** 2026-09-30

**Authors:** Yasuhiko Ago, Amali Karunathilaka, Jacky M. Guerrero-Vargas, Shunji Tomatsu

**Affiliations:** 1 Department of Biomedical Research, Nemours Children’s Health, Wilmington, DE, United States; 2 Department of Pediatrics, Thomas Jefferson University, Philadelphia, PA, United States; 3 Department of Biological Sciences, University of Delaware, Newark, DE, United States

**Keywords:** drug delivery, endochondral ossification, NPR-B agonist, peptide therapeutics, skeletal dysplasia, translational medicine

## Abstract

Disorders affecting cartilage and bone development frequently originate from disrupted endochondral ossification within growth-plate cartilage. Nonetheless, few therapeutic interventions directly target the local signaling pathways governing this process. This review explores the C-type natriuretic peptide (CNP)/natriuretic peptide receptor B (NPR-B) axis as a mechanism-based therapeutic approach addressing issues in cartilage and bone, including skeletal dysplasias, genetic short-stature conditions, and acquired ailments such as bone fractures. It summarizes the production and processing of CNP *in vivo*, elucidates how NPR-B activation induces cyclic guanosine monophosphate signaling in chondrocytes, and discusses how this pathway functionally antagonizes FGFR3-driven MAPK signaling in achondroplasia. Additionally, it traces the progression from native, rapidly cleared CNP peptides to engineered agonists optimized for enhanced stability, prolonged systemic exposure, or tissue retention, emphasizing exposure design. Clinically approved agents, such as vosoritide and navepegritide, exemplify how NPR-B agonism has become a viable treatment strategy for pediatric achondroplasia. Both U.S. approvals were accelerated, with final-height confirmation required. Emerging long-acting analogs, sustained-release prodrugs, matrix-binding constructs, Fc-fusion proteins, and elastin-like polypeptide fusion platforms demonstrate the potential for molecular design adaptations to meet various therapeutic objectives. The scope of potential applications now extends to hypochondroplasia, several non-achondroplasia genetic short-stature disorders, acquired growth-plate impairments, fracture healing, and osteoarthritis-related cartilage strategies. Overall, NPR-B agonism signifies a transition from systemic endocrine-driven growth promotion to the controlled modulation of skeletal growth and repair. Future developments will necessitate long-term evidence concerning final height, skeletal proportions, functional outcomes, joint health, quality of life, and safety.

## Graphical Abstract

**Figure:**
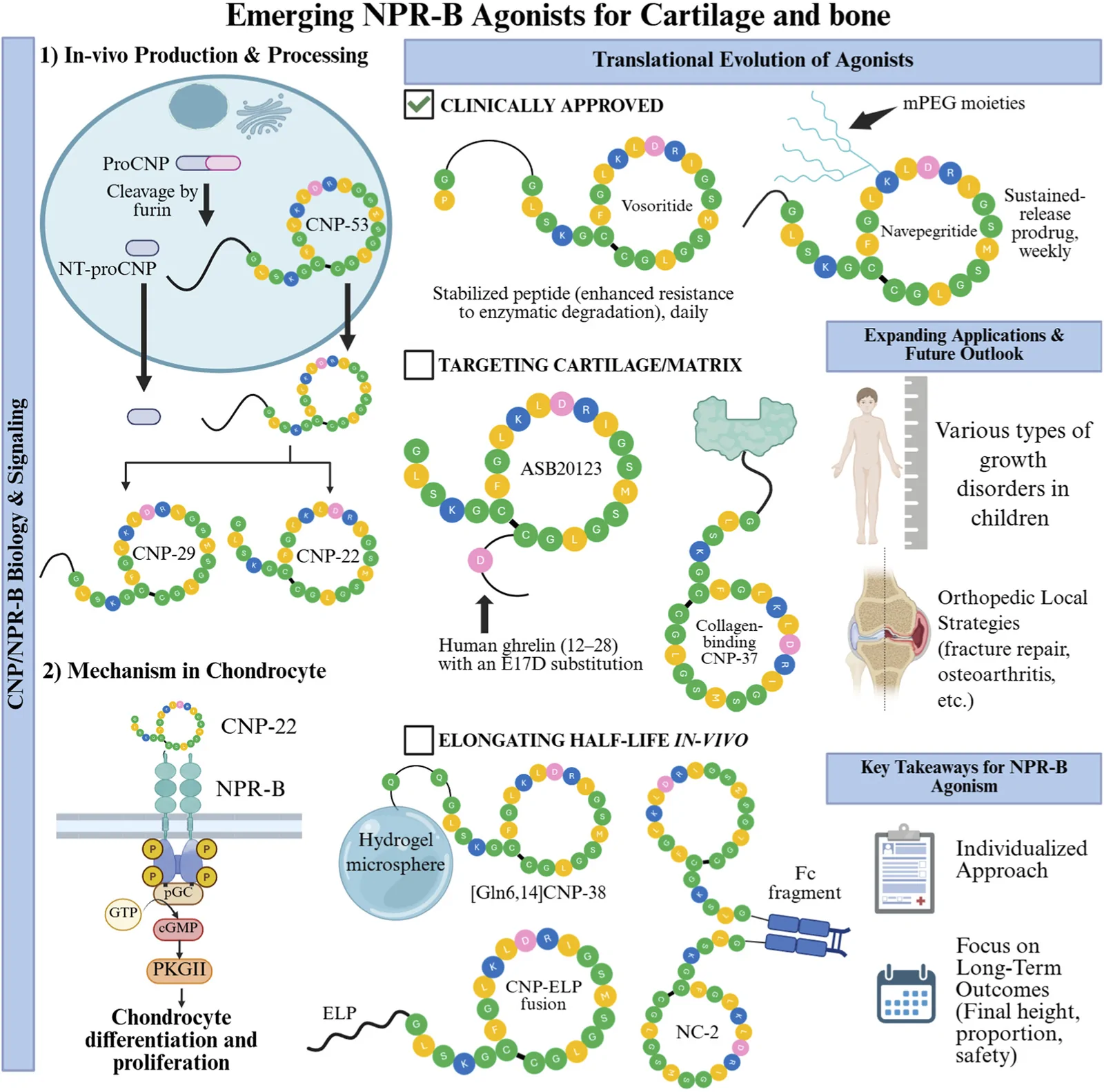

## 1 Introduction

Disorders of cartilage and bone growth remain challenging to treat because many originate in the growth plate, the specialized cartilage tissue where endochondral ossification enables longitudinal bone growth. In cases of skeletal dysplasias and certain genetic short-stature conditions, disrupted chondrocyte proliferation, hypertrophic differentiation, and extracellular matrix production can result not only in reduced stature but also in disproportionate skeletal development, craniofacial and skull-base deformities, spinal or limb complications, pain, and functional impairments (Maghnie et al., 2023; Yasoda et al., 2004). Conventional endocrine or orthopedic therapies are beneficial in specific contexts; however, they do not directly address the local signaling imbalance within growth-plate cartilage (Chikuda et al., 2004; Miyazawa et al., 2002; Potter et al., 2009; Yasoda et al., 2004).

C-type natriuretic peptide (CNP) and its receptor, natriuretic peptide receptor B (NPR-B), have been identified as a central regulator of growth-plate biology. The binding of CNP to NPR-B elevates intracellular cGMP levels and activates downstream signaling pathways that support the orderly maturation of chondrocytes and the process of endochondral bone formation (Miyazawa et al., 2002; Nakao et al., 2015; Potter et al., 2006; Potter et al., 2009; Yasoda et al., 2004). Genetic studies in humans have reinforced this concept: loss-of-function variants in *NPR2*, which encodes NPR-B, impair skeletal growth, whereas activating variants are associated with tall stature or skeletal overgrowth phenotypes (Badawi et al., 2023; Bartels et al., 2004; Chen et al., 2023; Hannema et al., 2013; Irfanullah et al., 2018; Jeong et al., 2026; Lauffer et al., 2020; Wei et al., 2025; Wu et al., 2022). These bidirectional findings from human genetics render NPR-B particularly compelling as a pharmacological target, given that both deficiency and excess of pathway activity manifest in human skeletal phenotypes.

The most advanced clinical entry point for NPR-B agonism has been achondroplasia. In this disorder, activating *FGFR3* variants suppress chondrocyte proliferation and differentiation, largely through activation of the MAPK pathway (Högler and Ward, 2020). CNP/NPR-B/cGMP signaling functionally antagonizes FGFR3-driven downstream MAPK signaling, providing a mechanistic rationale for using CNP-derived agonists rather than nonspecific growth-promoting agents (Lorget et al., 2012; Wendt et al., 2015; Yasoda et al., 2004). Preclinical administration of CNP and subsequent development of stabilized or sustained-release CNP analogs have translated this concept into clinically useful therapies, including once-daily and once-weekly systemic approaches for pediatric achondroplasia with open growth plates (Breinholt et al., 2019; Savarirayan et al., 2019; Savarirayan et al., 2020; Yasoda et al., 2009; Author Anonymous, 2026c). This trajectory highlights a key theme of the field: the challenge is not only to activate NPR-B, but also to control the duration, intensity, and tissue distribution of a normally short-lived peptide signal.

As the field matures, the potential scope of NPR-B agonism extends beyond achondroplasia. Conditions such as hypochondroplasia, partial *NPR2* deficiency, *NPPC*/CNP deficiency, certain Ras-MAPK-related disorders, *ACAN*-related short stature, glucocorticoid-associated growth impairment, and localized cartilage or bone repair have all been considered to varying extents (Boucher et al., 2022; Dauber et al., 2024; Dauber et al., 2026; Hirai et al., 2025; Inoue et al., 2019; Ono et al., 2013; Ueda et al., 2019). These prospective applications vary considerably. Pediatric growth disorders may necessitate sustained systemic exposure during a specific developmental window, whereas strategies for fracture repair or osteoarthritis may require local retention within matrix-rich tissues. Consequently, molecules targeting the same receptor may serve markedly different therapeutic purposes depending on their structure, pharmacokinetic profiles, and tissue-targeting properties.

This review examines emerging molecules that target the CNP/NPR-B axis as a strategy for treating cartilage and bone disorders. We first summarize how CNP is expressed and processed *in vivo* and then explain how activation of NPR-B regulates growth-plate chondrocytes and endochondral ossification. We next review how the NPR-B agonist strategy has evolved from native CNP biology to clinically approved and exposure-engineered agents. Finally, we compare each molecule based on its characteristic design features, including native reference ligands, neutral endopeptidase-resistant analogs, sustained-release prodrugs, long-acting systemic constructs, matrix-retained molecules, Fc-fusion proteins, and elastin-like polypeptide fusion platforms. By linking molecular design to biological purpose, this review aims to clarify both the promise and the current limitations of NPR-B agonism in skeletal medicine.

The distinctive contribution of this review is an exposure-design framework that follows each engineering intervention through linked consequences for molecular stability, pharmacokinetics, tissue distribution, pharmacodynamic pattern, dosing interval, and indication fit. Agents are therefore evaluated according to the delivery problem each design is intended to solve and the strength of the supporting evidence, rather than ranked by novelty or by indirect cross-trial comparisons.

## 2 The physiology of CNP in the skeletal system

### 2.1 How CNP is expressed and processed *in vivo*

The *NPPC* gene encodes human CNP. The gene is transcribed and translated to produce a precursor protein called preproCNP. This precursor is sequentially cleaved within the cell (Wu et al., 2003). The second cleavage by the enzyme furin splits proCNP into the mature, biologically active CNP-53 (53 amino acids) and an N-terminal fragment, NT-proCNP, which is not known to activate NPR-B and is widely used as a biomarker of CNP production (Ago et al., 2025; Wu et al., 2003). This mature CNP-53 and NT-proCNP are then released from neurons, glial cells, vascular endothelial cells, or chondrocytes in cartilage, including growth plates (Hodes and Lichtstein, 2014; Korostyshevskaya et al., 2016; Nakao et al., 2015; Ogawa et al., 1992; Sudoh et al., 1988; Suga et al., 1993). The N-terminus of CNP-53 is further cleaved in the extracellular space to form CNP-29 or CNP-22 (Ishizaka et al., 1992). Secreted CNP stimulates NPR-B on target cells to exert its biological functions, primarily in bone growth at growth plates and vascular homeostasis (Nakagawa and Nishikimi, 2022; Potter et al., 2006). Natriuretic peptide receptor C (NPR-C) contributes to clearance by binding and internalizing natriuretic peptides for intracellular degradation and receptor recycling, thereby reducing extracellular exposure (Hunt et al., 1994; Potter et al., 2006; Figure 1).

**FIGURE 1:**
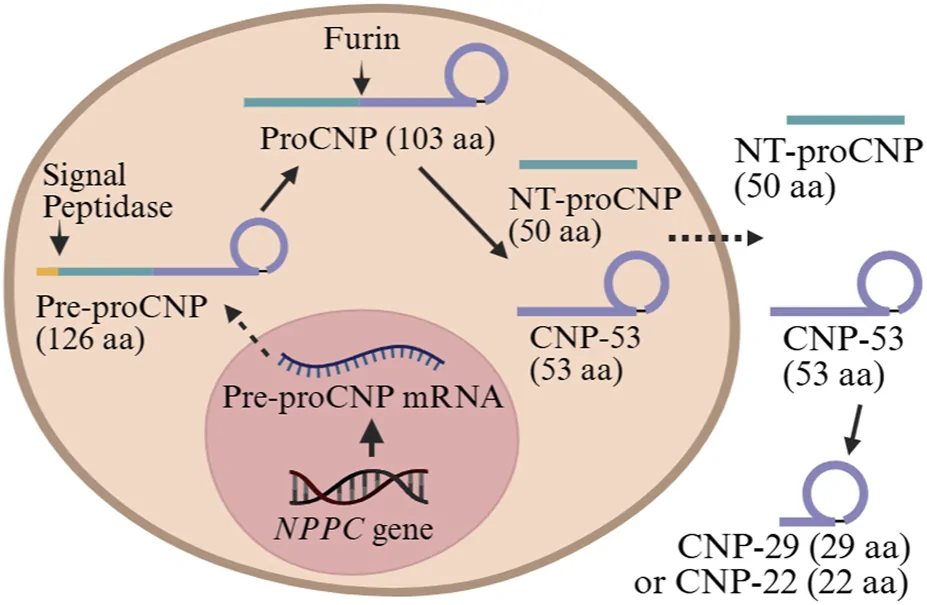
A schematic representation of the processing pathway of human pro-CNP. Human pro-CNP is initially synthesized as a 126-amino-acid prepropeptide. Following the removal of the signal peptide by signal peptidase, pro-CNP undergoes cleavage by furin, resulting in the formation of propeptide 1–50 (NT-proCNP) and CNP-53, both of which are subsequently secreted extracellularly. CNP-53 is further cleaved to form CNP-29 or CNP-22. This figure was created using https://BioRender.com.

### 2.2 The role of NPR-B: how NPR-B activation improves skeletal growth

The molecular identity of NPR-B was initially established by Chang et al. (1989), who identified and cloned the human natriuretic peptide receptor 2 (*NPR2*) gene and ascertained that it encodes the natriuretic peptide receptor B (NPR-B). At that time, the receptor was referred to as the ANP-B receptor (Chang et al., 1989).

Shortly following this cloning investigation, Koller et al. (1991) established the connection between NPR-B and its primary ligand by demonstrating that CNP specifically activates human NPR-B with significantly greater affinity than ANP or BNP (Koller et al., 1991).

These molecular findings were subsequently linked to skeletal biology by Bartels et al. (2004), who demonstrated that mutations in *NPR2*, the gene encoding NPR-B, compromise skeletal growth and result in acromesomelic dysplasia, Maroteaux type. This research established *NPR2* as a significant regulator of human skeletal development (Bartels et al., 2004).

Despite this functional and genetic evidence, to the best of our knowledge, no experimentally determined structure of human NPR-B (the protein encoded by *NPR2*) has been deposited in the Protein Data Bank. Nonetheless, the domain organization of NPR-B is thoroughly annotated: it is a single-pass transmembrane receptor guanylyl cyclase comprising an extracellular CNP-binding domain, a single transmembrane helix, and intracellular kinase-homology, oligomerization/helical, and guanylyl cyclase catalytic domains (Badawi et al., 2023; Bartels et al., 2004; Chen et al., 2023; Hannema et al., 2013; Irfanullah et al., 2018; Jeong et al., 2026; Khan et al., 2012; Knake et al., 2009; Lauffer et al., 2020; Wei et al., 2025; Wu et al., 2022). In the absence of a full-length experimental structure, homology modeling and AlphaFold-based predictions have been employed to estimate the structures of NPR-B domains or the entire NPR-B protein, particularly to interpret disease-associated *NPR2* variants (Badawi et al., 2023; Chen et al., 2023; Hannema et al., 2013; Irfanullah et al., 2018; Jeong et al., 2026; Lauffer et al., 2020; Wei et al., 2025; Wu et al., 2022).

To visualize this model-based structural inference, Figure 2 overlays the CNP-22-bound extracellular domain of NPR-C (PDB ID: 1JDP) with an AlphaFold 3-predicted structure of the NPR-B extracellular domain (NP_003986.2) in complex with CNP-22 (Abramson et al., 2024). The close structural agreement between the two extracellular domains suggests that NPR-B would recognize CNP-22 in a manner similar to NPR-C. However, this conclusion remains based on a predicted NPR-B structure, not an experimentally confirmed one. While it can be used to forecast completely novel agonist designs, experimental confirmation would always be mandatory.

**FIGURE 2:**
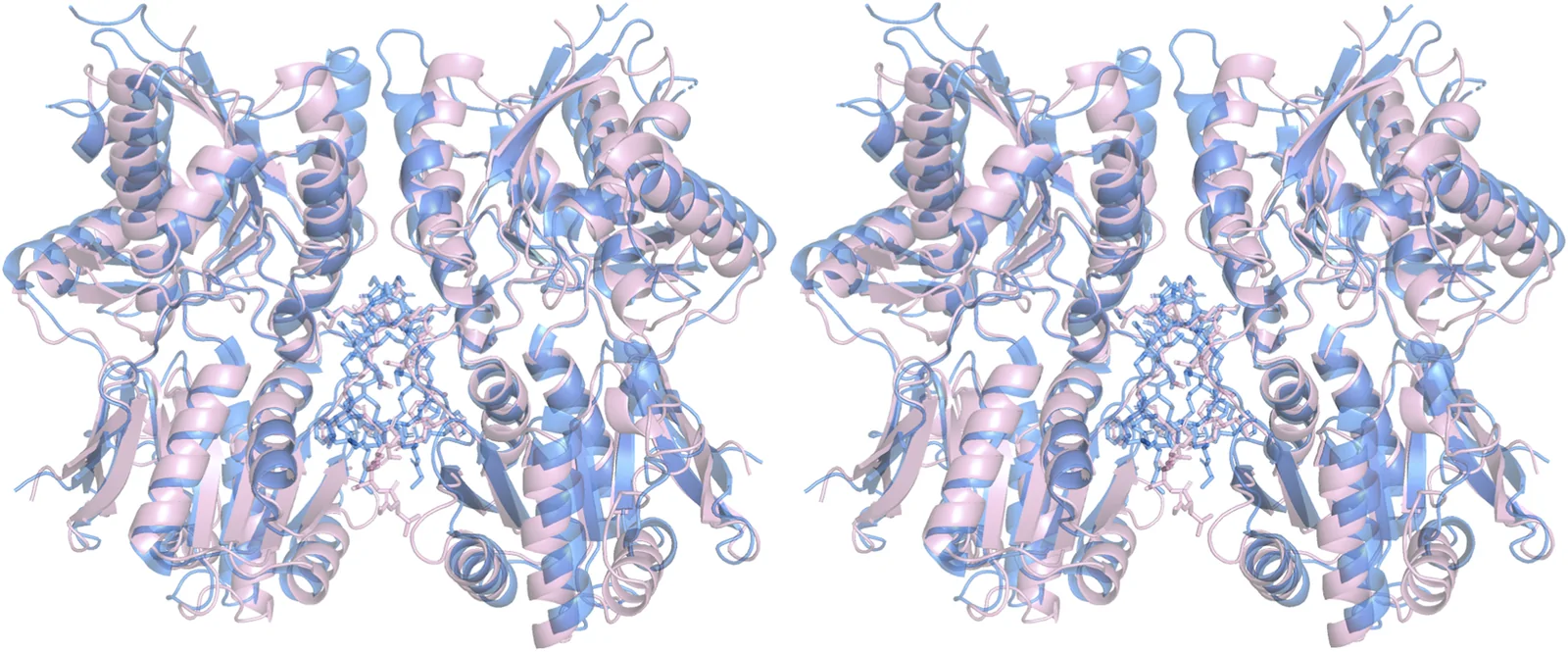
A predicted structure of NPR-B. A predicted structure of the extracellular domain of NPR-B (NP_003986.2; pink cartoon) with CNP-22 (pink sticks) is overlaid on NPR-C (blue cartoon) with CNP-22 (blue sticks) (PDB ID: 1JDP) in a cross-eye stereo view. The structures were superposed with a Cα RMSD of 1.484 Å over 606 aligned residues. The structural prediction of NPR-B was performed using AlphaFold 3, and its pTM score was 0.91 (Abramson et al., 2024). Two copies of the 436 amino acid residues, ranging from the 23rd to the 458th amino acid of NP_003986.2, as well as one amino acid sequence of CNP-22, were uploaded to the AlphaFold server. The templates were on up to 2/2/2025, and then the prediction was executed. The overlap was generated using PyMOL version 2.5.8. RMSD: root-mean-square deviation. pTM: predicted template modeling score.

While Figure 2 illustrates the anticipated interaction between NPR-B and the CNP-22 core, this structural model does not include the 31-residue N-terminal extension found in CNP-53. Functional investigations conducted in mouse astrocytes demonstrated that both CNP-22 and CNP-53 evoke broadly comparable NPR-B-mediated cGMP responses (Yeung et al., 1996). These results imply that the N-terminal extension of CNP-53 does not significantly hinder receptor activation. Nonetheless, this interpretation remains inferential for human NPR-B, as it is derived from functional data obtained from mouse cells rather than from direct experimental evidence involving the human receptor.

Mechanistically, following the binding of CNP to the extracellular domain of NPR-B, activation is transmitted through the single transmembrane segment to the intracellular guanylyl cyclase domain, which catalyzes the production of cyclic guanosine monophosphate (cGMP), thereby increasing intracellular cGMP levels (Potter et al., 2009). In growth-plate chondrocytes, cGMP activates cGMP-dependent protein kinase II (PKGII), leading to the phosphorylation of downstream regulators that coordinate chondrocyte proliferation, hypertrophic differentiation, and extracellular matrix synthesis (Chikuda et al., 2004; Potter et al., 2009). Collectively, these cellular effects support the orderly maturation of the growth plate, endochondral ossification, and longitudinal bone growth (Miyazawa et al., 2002; Potter et al., 2009; Yasoda et al., 2004).

## 3 Recent and past research on the NPR-B agonist strategy

Research concerning NPR-B agonism has progressed from foundational target validation to the development of clinically approved, exposure-optimized CNP analogs. Initial molecular, cartilage, and genetic studies shifted the view of CNP/NPR-B/cGMP signaling from a system studied mainly in cardiovascular and neuroendocrine contexts to a physiological regulator of growth-plate chondrocyte function, endochondral ossification, and longitudinal bone growth (Chusho et al., 2001; Hagiwara et al., 1994; Koller et al., 1991; Miyazawa et al., 2002; Nakao et al., 2015; Tamura et al., 2004; Yasoda et al., 1998). Human genetic research provides compelling support for this paradigm: loss-of-function variants in *NPR2* lead to severe skeletal growth deficiencies, whereas activating variants correlate with increased stature (Badawi et al., 2023; Bartels et al., 2004; Chen et al., 2023; Hannema et al., 2013; Irfanullah et al., 2018; Jeong et al., 2026; Lauffer et al., 2020; Wei et al., 2025; Wu et al., 2022). Concurrently, animal studies have demonstrated that CNP signaling enhances growth-plate activity and that chondrocyte-specific overexpression of CNP can mitigate the achondroplasia phenotype by functionally antagonizing growth-inhibitory MAPK signaling downstream of activated FGFR3 (Miyazawa et al., 2002; Yasoda et al., 2004). Accordingly, the initial therapeutic strategy was not merely to administer a universal growth factor but to potentiate an endogenous pathway that counteracts excessive growth-inhibitory MAPK signaling in chondrocytes.

The initial phase of this drug-focused strategy employed native CNP molecules to determine whether systemic activation of NPR-B could induce measurable skeletal growth. CNP-22, as the biologically authentic short form, demonstrated proof of concept that CNP administration can enhance endochondral bone growth in mouse models of achondroplasia (Yasoda et al., 2009). Nevertheless, native CNP-22 is rapidly eliminated via neutral endopeptidase and receptor-mediated clearance, necessitating continuous infusion during preclinical studies (Brandt et al., 1995; Brandt et al., 1997; Hunt et al., 1994; Yasoda et al., 2009). This pharmacokinetic limitation shifted the primary inquiry from whether NPR-B activation could promote bone growth to how to achieve sufficient, sustained, and safe NPR-B activation in pediatric populations. Consequently, CNP-53, the longer tissue-predominant form, was evaluated as a more persistent endogenous molecule (Ueda et al., 2019). It augmented skeletal growth in models of glucocorticoid-induced growth retardation and improved craniofacial morphology and foramen magnum size in CNP-deficient rats, suggesting that this approach could be relevant not only to lengthening limbs but also to skull-base and craniofacial development (Ueda et al., 2019; Yotsumoto et al., 2019).

The subsequent significant stage involved engineering native CNP biology into more pharmacologically favorable molecules. Vosoritide converted the concept into a viable chronic therapy through the utilization of a neutral endopeptidase-resistant CNP analog that sustains direct NPR-B agonism (Lorget et al., 2012; Wendt et al., 2015). In cases of achondroplasia, initial dose-finding and subsequent placebo-controlled clinical trials demonstrated increased annualized growth velocity, thereby supporting regulatory approval for pediatric patients with open epiphyses (Savarirayan et al., 2019; Savarirayan et al., 2020). More recently, research has advanced toward minimizing injection burden and optimizing systemic exposure. Navepegritide employs prodrug technology to release active CNP over a weekly interval and is presently an approved once-weekly treatment for pediatric achondroplasia in the United States (Breinholt et al., 2019; Author Anonymous, 2026c). BMN 333 is under development as another long-acting systemic CNP formulation (Author Anonymous, 2025a; Author Anonymous, 2025b; Hardy, 2025; Hardy and Friberg, 2026), whereas preclinical approaches such as [Gln6,14]CNP-38 and CNP-ELP fusions utilize chemical stabilization, depot release, or fusion technology to prolong exposure (JAMES & JAMES, 2017; Jowett et al., 2021; MacEwan and Chilkoti, 2014; Schneider et al., 2022). These developments illustrate that the field has transitioned from simple CNP replacement to exposure engineering. The relevant translational question is therefore not simply whether exposure is prolonged, but whether the resulting concentration-time profile matches the desired tissue, dosing interval, pharmacodynamic pattern, and safety margin.

The spectrum of diseases and conditions considered within this strategic framework has expanded over time. Clinical development efforts have been particularly robust for achondroplasia, owing to its mechanism closely aligning with the pharmacology of NPR-B agonism: activating *FGFR3* variants suppress chondrocyte proliferation and differentiation via MAPK, whereas CNP/NPR-B/cGMP signaling functionally antagonizes FGFR3-driven downstream MAPK signaling (Lorget et al., 2012; Wendt et al., 2015; Yasoda et al., 2004). This rationale naturally extends to hypochondroplasia, another skeletal dysplasia associated with *FGFR3*. A phase 2 study of vosoritide in children with hypochondroplasia demonstrated increased growth velocity, and further clinical development remains underway (Dauber et al., 2024). The same pathway has also stimulated investigations into several other genetic conditions characterized by short stature, extending beyond achondroplasia. These include disorders in which CNP supply is deficient, such as *NPPC*/CNP deficiency; conditions with diminished but not absent NPR-B signaling, exemplified by heterozygous or partial *NPR2* deficiency; Ras/MAPK pathway-related disorders, including Noonan syndrome, cardio-facio-cutaneous syndrome, and neurofibromatosis type 1; as well as extracellular-matrix disorders such as *ACAN*-related short stature (Boucher et al., 2022; Dauber et al., 2026; Inoue et al., 2019; Jeong et al., 2026; Ono et al., 2013).

Other conditions have been examined in a more exploratory manner. Turner syndrome and *SHOX* deficiency are currently being assessed through basket-style clinical programs, particularly in pediatric patients experiencing insufficient growth during or after human growth hormone therapy (ClinicalTrials.gov Identifier: NCT06668805). The mechanistic rationale in these contexts may be less direct than in disorders related to *FGFR3*-, *NPPC*/*NPR2*-, or Ras/MAPK pathways; therefore, evidence should be evaluated independently for each condition. Additionally, NPR-B agonism has been investigated in acquired or localized orthopedic settings. Preclinical studies using CNP-53 in glucocorticoid-induced growth retardation suggest a potential role in medication-associated growth impairment, while the collagen-binding CNP-37 has expanded this concept to localized delivery for fracture repair and osteoarthritis models (Hirai et al., 2025; Ueda et al., 2019). The latter application differs from systemic pediatric growth therapy, as it seeks to harness CNP effects on chondrogenesis, osteogenesis, and matrix-rich tissue repair rather than promoting overall height increase.

The cumulative history of NPR-B agonist research exemplifies a progressive expansion along two primary dimensions: initially, from the validation of growth-plate targets to the development of approved treatments for achondroplasia; and subsequently, from the utilization of native, short-lived peptides to the creation of engineered molecules specifically designed to optimize stability, dosing intervals, and tissue distribution. This developmental sequence elucidates why each agent should be evaluated based on its principal design features rather than solely by chronological emergence or clinical phase. Consequently, the following section provides a summary of each molecule, emphasizing the structural or formulation strategy that distinguishes it: native reference ligands, neutral endopeptidase-resistant analogs, sustained-release prodrugs, long-acting systemic constructs, cartilage- or collagen-retained molecules, Fc-fusion proteins, or ELP-based fusion approaches. Accordingly, the agent-level analysis below follows a common sequence: structural intervention, exposure consequence, pharmacodynamic implication, dosing strategy, intended application, and evidence tier.

## 4 Agent-by-agent analysis: linking molecular design to exposure and intended use

### 4.1 CNP-22

CNP-22 is the 22-amino-acid endogenous short form of CNP and constitutes the predominant circulating form of the peptide (Moyes and Hobbs, 2019). It specifically binds to and activates NPR-B/GC-B, thereby inducing cGMP generation, so it is the native benchmark agonist for this signaling pathway (Potter et al., 2009; Yeung et al., 1996). In the context of skeletal dysplasia research, exogenous CNP-22 provided the early proof of concept that systemic CNP treatment can ameliorate impaired endochondral bone growth in achondroplasia models (Yasoda et al., 2009). Its primary limitation as a therapeutic candidate is its rapid clearance and poor *in vivo* persistence, necessitating continuous intravenous infusion for effective preclinical application rather than convenient intermittent dosing (Yasoda et al., 2009). Consequently, CNP-22 is best regarded as the biologically authentic reference ligand, albeit with poor pharmacokinetic properties that are not ideal for drug development. In humans, the plasma half-life of CNP-22 is approximately 2.6 min; NEP degradation and NPR-C-mediated internalization explain why effective skeletal proof-of-concept studies required continuous infusion (Brandt et al., 1995; Brandt et al., 1997; Hunt et al., 1994; Yasoda et al., 2009). Thus, its principal translational role is as a pharmacodynamic benchmark rather than a practical intermittent therapy (Table 1; Figure 3).

**TABLE 1:** Molecular design, exposure strategy, and evidence base of NPR-B agonists.

Agent	Native or engineered?	Structural modification	Exposure/targeting strategy	Development stage/primary evidence	Design-to-use interpretation
CNP-22	Native endogenous 22-amino-acid CNP	No engineering: it is the predominant circulating form of CNP.	It is rapidly cleared; human half-life is about 2.6 min, so efficacious achondroplasia-model work generally needed continuous intravenous infusion. Clearance reflects both NEP degradation and NPR-C-mediated internalization	Preclinical proof-of-concept for skeletal growth rescue. Primary evidence: human pharmacokinetics plus peer-reviewed preclinical skeletal studies	The native benchmark that proved NPR-B activation can work, but not a practical chronic drug scaffold
CNP-53	Native endogenous 53-amino-acid CNP	The longer native form and the form that is predominant in tissues	More tissue-associated than CNP-22; it was used in later systemic studies and improved craniofacial growth and foramen magnum size in CNP-knockout rats. A formal human CNP-53 half-life has not been reported; the persistence advantage is relative and mainly preclinical	Preclinical native comparator. Primary evidence: peer-reviewed rodent studies	A tissue-predominant native form, especially useful when craniofacial or skull-base growth is of interest (Hirota et al., 2018; Yotsumoto et al., 2019)
Vosoritide (BMN 111; VOXZOGO)	Engineered peptide analog	A 39-amino-acid analog made from the 37 C-terminal residues of human CNP-53 plus an N-terminal Pro-Gly extension for neutral endopeptidase resistance	Direct systemic NPR-B agonist given once daily by subcutaneous injection; it functionally antagonizes FGFR3-driven downstream MAPK signaling. Mean pediatric half-life: 27.9 min; once-daily subcutaneous dosing	FDA-approved for pediatric achondroplasia with open epiphyses. Sponsor-announced phase 3 topline results in hypochondroplasia are positive; peer-reviewed publication is pending. Achondroplasia phase 3 placebo-adjusted annualized growth velocity: +1.57 cm/year (95% CI, 1.22–1.93). Primary evidence: peer-reviewed randomized clinical trial	The first FDA-approved stabilized CNP analog; the Pro-Gly modification supports daily dosing rather than long-interval depot exposure
Navepegritide (TransCon CNP; YUVIWEL)	Engineered prodrug	Active CNP moiety is identical to human CNP residues 89–126 and is transiently linked to two branched 20 kDa mPEG groups through a TransCon linker	After subcutaneous dosing, it auto-cleaves, providing continuous systemic CNP exposure over a weekly interval; the released CNP has the same receptor-binding affinity and activity as endogenous CNP. Apparent half-lives: 6.7 days for the prodrug and 5.3 days for released CNP; once-weekly subcutaneous dosing	FDA-approved in 2026 for pediatric patients 2 years of age and older with achondroplasia with open epiphyses. Accelerated approval based on annualized growth velocity; placebo-adjusted effect: +1.49 cm/year (95% CI, 1.05–1.93). Primary evidence: peer-reviewed randomized clinical trial	A cleavable prodrug that converts PEG-associated residence time into continuous weekly CNP exposure
BMN 333	Engineered long-acting CNP program	Public sources describe a long-acting CNP program, but the amino acid sequence and carrier architecture have not been disclosed	Sponsor-reported phase 1 healthy-adult data support sustained released-CNP exposure compatible with once-weekly dosing	Phase 2/3 clinical trial (NCT07441876) is enrolling participants as of August 2026. Primary evidence: the trial registry plus sponsor and conference disclosures. No peer-reviewed pediatric efficacy data are available	A late-stage long-acting clinical program whose molecular architecture and comparative clinical efficacy remain insufficiently characterized in public peer-reviewed sources
ASB20123	Engineered chimeric peptide	A 39-amino-acid CNP/ghrelin chimera: CNP–22 fused to ghrelin (12–28, E17D)	It showed longer plasma half-life and higher cartilage distribution than CNP-22; the ghrelin tail’s basic/BX7B motif may promote extracellular-matrix retention, and subcutaneous infusion outperformed bolus dosing. Design chain: larger/basic ghrelin-derived extension - > longer plasma persistence and increased cartilage distribution - > cartilage-preferring systemic exposure	Preclinical. Primary evidence: peer-reviewed mouse and rat studies	A cartilage-preferring systemic chimera, with both exposure-extension and matrix-retention features (Morozumi et al., 2019)
Microsphere [Gln6,14]CNP-38 conjugates	Engineered stable analog	Built from the 38 C-terminal residues of human CNP-53 with Asn→Gln substitutions at positions 6 and 14 to prevent deamidation	The peptide core was optimized for depot formulations; microsphere conjugates were designed for weekly or monthly release and showed long terminal half-lives in mice. *In vitro* degradation was approximately 2.7% per week; mouse terminal half-lives were approximately 200 h (weekly depot) and 600 h (monthly depot)	Preclinical. Primary evidence: peer-reviewed preclinical study; human pharmacokinetics are unavailable	A chemically stabilized CNP analog designed for long-interval depot dosing (Schneider et al., 2022)
Collagen-binding CNP-37	Engineered matrix-targeted fusion	A collagen-binding domain at the N-terminus and CNP-37 at the C-terminus, connected by a Gly-Gly-Ser-Pro-Gly spacer	Designed to stay in collagen-rich tissue; a single dose produced sustained bone elongation *ex vivo*, and local persistence/benefit was shown in fracture and osteoarthritis models. Articular-cartilage detectability was demonstrated 3 days after intra-articular injection in mice	Preclinical localized orthopedic construct. Primary evidence: peer-reviewed *ex vivo* and mouse studies	A local-delivery, tissue-retained CNP strategy (Hirai et al., 2025)
NC-2	Engineered recombinant Fc-fusion	Human CNP-22 preceded by six propeptide residues and fused through a (GGGGS)2 linker to human IgG1 Fc	A systemic exposure-engineered recombinant; in Nf1Col2^−/−^ mice, daily subcutaneous dosing reduced phosphorylated ERK and rescued growth retardation. Reported mouse half-life: approximately 20 h; daily subcutaneous dosing was used in the NF1 model	Preclinical, mainly in NF1 growth-plate dysplasia rather than mainstream achondroplasia development. Primary evidence: peer-reviewed mouse studies; additional variants are described in patents	Best thought of as an Fc-fusion biologic with an extended half-life, not a simple peptide analog or a local CNP construct (Ono et al., 2013)
CNP-ELP fusion	Engineered recombinant fusion protein	CNP is recombinantly fused to an elastin-like polypeptide (ELP), an elastin-derived biopolymer designed to retain peptide bioactivity while increasing molecular size and stability	ELP provides systemic half-life extension rather than specific cartilage targeting: after subcutaneous injection, thermoresponsive depot formation and slow release can prolong exposure; CNP-ELP was reported to remain detectable for up to 24 h and to increase growth velocity in mice (reported in a conference abstract rather than a full peer-reviewed skeletal efficacy article)	Preclinical/proof-of-concept; strongest evidence is patent disclosure and a conference abstract, not a full peer-reviewed skeletal efficacy article	A proposed long-acting CNP delivery platform, but its skeletal evidence base is less mature and less transparent than those of sequence-defined CNP analogues

**FIGURE 3:**
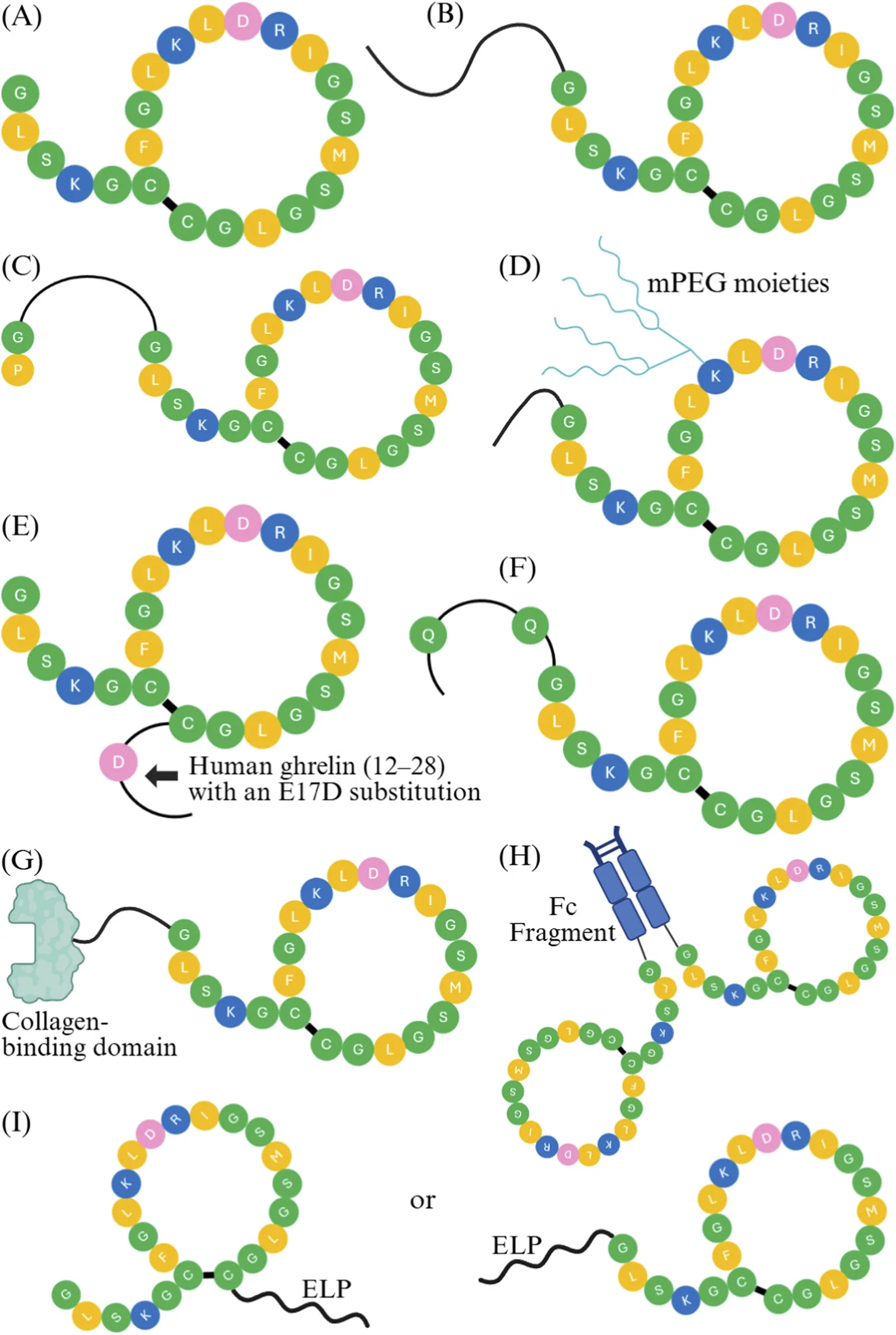
Schematic illustrations of each agent except BMN 333. **(A)** CNP-22, **(B)** CNP-53, **(C)** Vosoritide, **(D)** Navepegritide **(E)** ASB20123, **(F)** [Gln6,14]CNP-38, **(G)** Collagen-binding CNP-37, **(H)** NC-2, **(I)** CNP-ELP fusion. All illustrated molecules contain the CNP-22 receptor-binding core. This figure was created using BioRender.com.

### 4.2 CNP-53

CNP-53 represents the 53-amino-acid endogenous long form of CNP and is the predominant active form at the tissue level (Potter et al., 2009; Yeung et al., 1996). It can activate NPR-B with pharmacology broadly similar to CNP-22; however, it persists longer *in vivo* due to its reduced sensitivity to neutral endopeptidase (NEP) (Espiner et al., 2019; Ueda et al., 2019). This pharmacokinetic advantage has led investigators to select CNP-53 specifically for subsequent skeletal studies, considering it a more practical native molecule than CNP-22 (Espiner et al., 2019; Ueda et al., 2019). In CNP-deficient rats, exogenous CNP-53 accelerated craniofacial skeletogenesis and improved craniofacial morphology and foramen magnum size, rendering it particularly attractive for dysplasias in which skull-base growth is as crucial as limb development (Yotsumoto et al., 2019). Compared to CNP-22, CNP-53 remains a native CNP molecule but offers enhanced tissue-relevant persistence. A formal human plasma half-life for CNP-53 has not been reported; therefore, its persistence advantage should be interpreted as a relative, mainly preclinical observation rather than a quantified human pharmacokinetic advantage (Espiner et al., 2019; Ueda et al., 2019).

### 4.3 Vosoritide (BMN 111; VOXZOGO)

Vosoritide is a 39-amino-acid recombinant human CNP analog made from the 37 C-terminal amino acids of human CNP-53 and an N-terminal Pro-Gly extension that enhances resistance to NEP degradation (D. M. Galetaki and Dauber, 2024; Lorget et al., 2012). It remains a direct NPR-B agonist and functionally antagonizes hyperactive FGFR3-driven downstream MAPK signaling, thereby promoting chondrocyte proliferation, differentiation, and linear bone growth (Chan et al., 2022; Lorget et al., 2012). Preclinical studies demonstrated that BMN 111 corrected multiple features of FGFR3-driven dwarfism, including growth-plate abnormalities and various skeletal deformities in mouse models (Lorget et al., 2012; Wendt et al., 2015). Clinically, it became the first approved therapy targeting the CNP pathway for achondroplasia and is administered via once-daily subcutaneous injection. Favorable outcomes were also observed in hypochondroplasia during a Phase II clinical trial (Dauber et al., 2024). The phase III study NCT06455059 has reported sponsor-announced positive topline results in hypochondroplasia; peer-reviewed publication is pending (Author Anonymous, 2026a). Vosoritide is distinguished by a minimal, rational sequence alteration that preserves the native biology of CNP while enabling feasible chronic outpatient dosing (Chan et al., 2022). The Pro-Gly extension extends exposure sufficiently for once-daily dosing, but the mean half-life in children remains 27.9 min (Chan et al., 2022). In the pivotal phase 3 trial, the placebo-adjusted increase in annualized growth velocity at week 52 was 1.57 cm/year (95% CI, 1.22–1.93) (Savarirayan et al., 2020). This is a within-trial estimate and should not be used for indirect superiority comparisons with other agents.

### 4.4 Navepegritide (TransCon CNP; YUVIWEL)

Navepegritide is a prodrug of active CNP, wherein a CNP(89–126)/CNP-38 payload is linked via a cleavable TransCon linker to two branched 20-kDa mPEG moieties (Breinholt et al., 2019; Author Anonymous, 2026c). Following subcutaneous administration, the linker auto-cleaves, ensuring continuous systemic exposure to the released CNP throughout the weekly dosing interval, with predicted mean apparent half-lives of approximately 6.7 days for navepegritide and 5.3 days for the released CNP (Author Anonymous, 2026c). The released CNP demonstrates identical receptor-binding affinity and activity to that of endogenous CNP, binds to NPR-B, elevates cGMP levels, and functionally antagonizes FGFR3-driven downstream MAPK signaling. Clinically, it is a once-weekly therapeutic option and received FDA approval in 2026 for pediatric patients with achondroplasia who have open epiphyses (Author Anonymous, 2026c). Its defining exposure feature is continuous release of active CNP across the weekly dosing interval rather than a short post-injection concentration pulse. In the pivotal APPROACH trial, the least-squares mean difference in annualized growth velocity versus placebo was 1.49 cm/year (95% CI, 1.05–1.93) at 52 weeks (Savarirayan et al., 2026). FDA approval was granted under the accelerated approval pathway on the basis of annualized growth velocity; a postmarketing study is required to verify benefit on final adult height and to evaluate disproportionate growth (Author Anonymous, 2026c). These data establish efficacy versus placebo within that trial, but they do not establish superiority over vosoritide because the trials differed in study design, endpoints, and patient populations.

### 4.5 BMN 333

As of August 2026, BMN 333 is an enrolling registration-enabling phase 2/3 program that compares once-weekly BMN 333 with once-daily vosoritide in treatment-naive children with achondroplasia (NCT07441876). Sponsor and conference disclosures from a phase 1 single-ascending-dose study in healthy adults report sustained released-CNP exposure compatible with weekly dosing and acceptable short-term tolerability (Mitchell, 2026), but no peer-reviewed pediatric efficacy data are available. The specific amino acid sequence and carrier architecture remain undisclosed in public sources. Accordingly, BMN 333 should be interpreted as a long-acting clinical program supported principally by registry and sponsor-disclosed evidence rather than as a fully characterized sequence-defined analog (Author Anonymous, 2026a).

### 4.6 ASB20123

ASB20123 is a 39-amino-acid CNP/ghrelin chimera composed of the CNP-22 sequence fused to the 17-amino-acid C-terminal fragment of human ghrelin (12–28) with an E17D substitution (Morozumi et al., 2019). It was engineered to be approximately twice the size of CNP-22, thereby enhancing biokinetics while maintaining full NPR-B agonist activity (Morozumi et al., 2017; Morozumi et al., 2019). Compared with CNP-22, ASB20123 exhibited a prolonged plasma half-life and increased cartilage distribution after subcutaneous administration in mice (Morozumi et al., 2019). The authors suggested that the ghrelin-derived basic-residue cluster and the BX7B motif facilitate the retention of the peptide within the extracellular matrix of the growth plate (Morozumi et al., 2019). Multiple subcutaneous administrations demonstrated dose-dependent stimulation of skeletal growth in rats; furthermore, continuous subcutaneous infusion was more efficacious than bolus injection at equivalent doses. In summary, the principal design concept of ASB20123 is to achieve cartilage-preferring exposure while preserving direct NPR-B agonist activity (Morozumi et al., 2019). The modification-to-exposure logic is therefore: the ghrelin-derived extension increases molecular size and introduces basic residues, these features increase plasma persistence and cartilage distribution, and the resulting profile is intended for cartilage-preferring systemic exposure. Evidence remains limited to peer-reviewed preclinical studies.

### 4.7 Microsphere [Gln6,14]CNP-38 conjugates

[Gln6,14]CNP-38 is a stable long-acting CNP analog constructed from the 38 C-terminal amino acids of human CNP-53, featuring Asn→Gln substitutions at positions 6 and 14 to inhibit degradative deamidation (Schneider et al., 2022). The molecule has been reported to exhibit high activity and stability, with stability testing conducted by the authors—incubation over 4 weeks at pH 7.4 and 37 °C—indicating approximately 2.7% degradation per week (Schneider et al., 2022). Furthermore, the same study conjugated [Gln6,14]CNP-38 to a 60 μm tetra-polyethylene glycol hydrogel microsphere for once-weekly or once-monthly subcutaneous administration (Schneider et al., 2022). After subcutaneous administration in mice, the released [Gln6,14]CNP-38 demonstrated extended terminal half-lives of approximately 200 and 600 h, respectively (Schneider et al., 2022). Both formulations produced skeletal growth effects in the study’s mouse model, including direct experimental comparisons with daily vosoritide (Schneider et al., 2022). The primary innovation of [Gln6,14]CNP-38, therefore, resides in its chemical stabilization of the peptide itself, coupled with its suitability for long-interval depot delivery. This peer-reviewed evidence is preclinical; the quantitative half-lives are mouse terminal half-lives and should not be extrapolated directly to humans.

### 4.8 Collagen-binding CNP-37 (CBD-CNP)

CBD-CNP is a synthetic fusion protein wherein a collagen-binding domain is positioned at the N-terminus and CNP-37 at the C-terminus, connected by a Gly-Gly-Ser-Pro-Gly spacer (Hirai et al., 2025). Despite incorporating the targeting domain, it retains the ability to bind NPR-B and suppress FGF2-induced ERK1/2 signaling, demonstrating that the collagen-binding module does not negate CNP bioactivity (Hirai et al., 2025). Its primary purpose is to facilitate local matrix retention within collagen-rich tissues. In *ex vivo* fetal tibiae, a single administration resulted in sustained bone elongation, whereas a single dose of CNP-22 did not (Hirai et al., 2025). In mouse models of fracture and osteoarthritis, it demonstrated prolonged local presence, enhanced callus bone mineral content and bone volume, and remained detectable with 3,3′-diaminobenzidine staining in articular cartilage 3 days after intra-articular injection (Hirai et al., 2025). This construct is primarily designed for local matrix retention, making it a localized orthopedic CNP strategy rather than an analog intended to maximize systemic exposure. The design-to-use link is direct: collagen binding increases local residence, prolonged local residence extends pharmacodynamic activity, and the intended application shifts from systemic height promotion to focal cartilage or bone repair. The evidence is peer-reviewed but remains preclinical.

### 4.9 NC-2

NC-2 is a recombinant Fc-CNP fusion constructed from the human CNP-22 sequence plus six upstream propeptide residues, linked via a (GGGGS)2 glycine-rich spacer to the C-terminus of human IgG1 Fc, which has achieved a half-life of 20 h in mice (Ono et al., 2013). This Fc-fusion architecture markedly distinguishes it from simple peptide analogs such as vosoritide or [Gln6,14]CNP-38 (Ono et al., 2013; Philippe & Florent, 2013). In a published study on NF1 growth plates, recombinant NC-2 was injected daily and subcutaneously from birth for 18 days, resulting in enhanced bone growth in Nf1Col2−/− mice (Ono et al., 2013). A subsequent study concluded that NC-2 ameliorated growth retardation in chondrocyte-specific NF1-deficient mice by decreasing phosphorylated ERK levels in the growth plate (Inoue et al., 2019; Ono et al., 2013). Patent disclosures reveal that the broader NC-2 platform has been diversified into variants including NC2B, D10-NC2, NC2-KGANKK, NC2-KGANQK, and NC2-CNP53mut2 (Philippe and Florent, 2013). NC-2 is best characterized as an Fc-fusion–based exposure-engineering platform for which additional sequence variants have been disclosed in patents. Nonetheless, the publicly available evidence of efficacy remains preclinical and focuses on NF1-associated growth-plate dysplasia rather than on traditional drug development for achondroplasia. The 20 h half-life and growth effects were measured in mice; efficacy evidence is peer-reviewed and preclinical, whereas several platform variants are described only in patent disclosures.

### 4.10 CNP-ELP fusion

The fusion of elastin-like polypeptide (ELP) represents a potential platform for extending the half-life of peptide therapeutics. This platform has been investigated clinically using pemziviptadil/PB1046, a Vasoactive Intestinal Peptide (VIP)-ELP fusion, thereby providing clinical precedent for ELP-based peptide delivery outside the CNP field (ClinicalTrials.gov: NCT03315507, NCT03795428) (MacEwan and Chilkoti, 2014). ELPs are engineered protein chains derived from human elastin. They possess the capability to extend the half-life of therapeutic peptides via: (1) Depot formation—ELPs undergo a temperature-dependent phase transition that facilitates aggregation and the formation of a subcutaneous depot, enabling slow release; (2) Reduced renal clearance—ELP fusion increases the molecular size, consequently diminishing renal filtration and prolonging circulation time; (3) Protection from proteolysis—ELP fusion provides a protective barrier shielding peptides from proteolytic enzymes (MacEwan and Chilkoti, 2014).

PhaseBio Pharmaceuticals secured a patent for ELP Fusion proteins, with the patent application listing CNP among numerous peptides that can be fused to ELPs. The patent included experimental data demonstrating that CNP can be successfully fused to ELPs, thereby maintaining biological activity while enabling controlled release and improved pharmacokinetic properties (James and James, 2017). A conference abstract reported that CNP-ELP remained detectable in circulation for up to 24 h after a single injection and increased growth velocity in mice, consistent with prolonged NPR-B agonist activity (Jowett et al., 2021). Although Jowett et al. (2021) provided proof-of-concept that ELP fusion can convert short-lived peptides such as CNP into long-acting therapeutic agents, comprehensive *in vivo* efficacy data remain limited at present. Because the skeletal evidence is supported mainly by a patent and a conference abstract, CNP-ELP should be regarded as proof-of-concept rather than a clinically established CNP program.

## 5 Future perspectives of this NPR-B agonist strategy

The subsequent phase of NPR-B agonist research should define what this strategy is expected to modify clinically. For achondroplasia, the increase in annualized growth velocity has served as the most pragmatic early endpoint; however, it is insufficient to capture the full therapeutic value of long-term treatment. In the United States, vosoritide and navepegritide were initially approved under the accelerated approval pathway on the basis of annualized growth velocity, with confirmatory assessment of final adult height; the navepegritide postmarketing requirement also specifies evaluation of disproportionate growth (Author Anonymous, 2026b). Future research should consider assessing final adult stature, body proportionality, limb alignment, spinal and foramen magnum complications, sleep-related and respiratory outcomes, physical function, pain levels, the need for orthopedic or neurosurgical interventions, and outcomes reported by patients and their families. Such an expanded set of endpoints is essential, given that CNP/NPR-B agonism aims to mitigate an underlying disease mechanism within the growth plate, rather than solely to promote height increase (Breinholt et al., 2019; Chan et al., 2022; Savarirayan et al., 2019; Savarirayan et al., 2020; Author Anonymous, 2026c). Because the pivotal vosoritide and navepegritide studies enrolled different age ranges and used different protocols, their placebo-adjusted annualized-growth-velocity estimates should be interpreted within each trial rather than as a head-to-head comparison (Savarirayan et al., 2020; Savarirayan et al., 2026).

Expansion beyond achondroplasia represents a significant future trajectory; however, the robustness of the biological rationale varies across different conditions. The most straightforward extension pertains to other FGFR3-related skeletal dysplasias, particularly hypochondroplasia, for which vosoritide has already shown increased growth velocity in a phase 2 study (Dauber et al., 2024). Some Ras-MAPK-related disorders, along with *ACAN* deficiency and partial *NPR2* deficiency, are also rational candidates because they influence growth-plate signaling pathways that may converge, either directly or indirectly, with CNP/NPR-B/cGMP activity (Dauber et al., 2026). More severe FGFR3-related conditions, such as severe achondroplasia with developmental delay and acanthosis nigricans (SADDAN) or rare long-term survivors of thanatophoric dysplasia, may also be considered conceptually; however, current evidence for thanatophoric dysplasia type 1 is limited to a single case report and should therefore be regarded as hypothesis-generating rather than as evidence of efficacy (Montero-Lopez et al., 2025). Mucopolysaccharidoses (MPS) constitute another promising area of exploration. The systemic expression of CNP improved the skeletal symptoms of MPS VII model mice, particularly when combined with the systemic expression of Gusb gene (Kashiwagi et al., 2022; Yamashita et al., 2020). AAV-mediated NPPC expression induced bone growth in an MPS IVA mouse model, even without supplying the deficient enzyme N-acetylgalactosamine-6-sulfate-sulfatase (Rintz et al., 2023). These positive results at the preclinical stage paved the way for the Phase I/II clinical trial of vosoritide for MPS IVA and VI patients (ClinicalTrials.gov Identifier: NCT05845749). Although the results have not yet been peer-reviewed, the preliminary data from NCT05845749 seem promising (Fung et al., 2026; Lynch et al., 2026; Perdomo-Luna et al., 2026).

A second potential approach involves treating acquired or inflammatory growth-plate impairment. Preclinical investigations with CNP-53 indicate that CNP signaling can enhance glucocorticoid-induced impairment of skeletal growth (Ueda et al., 2019). Furthermore, overexpression of chondrocyte CNP has been shown to protect against growth retardation and articular cartilage damage in a murine model of inflammatory arthritis (Bükülmez et al., 2014). These findings suggest that NPR-B agonism could be explored in carefully defined pediatric subpopulations with chronic inflammatory disease, prolonged glucocorticoid exposure, or other conditions that lead to secondary suppression of growth-plate activity. Such applications necessitate rigorous disease-specific evaluation, given that growth failure associated with acquired diseases is often multifactorial, involving factors such as inflammation, nutrition, endocrine function, medication exposure, and mechanical influences. It should be noted that NPR-B agonism is not expected to solely rectify all causes of impaired growth.

The strategy may also expand from systemic pediatric growth therapy to localized cartilage and bone repair. Mature articular cartilage, fracture callus, and osteochondral defects are confined tissues or problems; consequently, the optimal pharmacological design for these conditions may differ significantly from that used to promote height gain in pediatric populations. Collagen-binding CNP-37 exemplifies this principle by utilizing matrix retention to extend local activity within collagen-rich tissues, demonstrating preclinical efficacy in fracture repair and osteoarthritis models (Hirai et al., 2025). Previous research in murine models with persistently elevated circulating CNP has also indicated enhanced bone turnover and potential acceleration of fracture healing (Kondo et al., 2015). These findings advocate further investigation into local or tissue-retained NPR-B agonists for treating delayed fracture union, nonunion, osteochondral repair, early osteoarthritis, and cartilage tissue engineering. Future development of localized NPR-B agonist strategies may also benefit from integration with broader regenerative-medicine approaches, including mesenchymal stromal/stem-cell-based cartilage and bone repair and biomaterial scaffolds or functionalized delivery platforms that enhance local retention and controlled release. In a directly relevant preclinical example, CNP gene-modified bone marrow mesenchymal stem cells delivered on a chitosan/silk-fibroin scaffold enhanced cartilage matrix formation and repair in a rat full-thickness defect model; however, this cell/gene-delivery strategy is distinct from local administration of a defined NPR-B agonist (Yang et al., 2019). Nonetheless, it is imperative to distinguish these applications in adults or localized orthopedic settings from systemic growth-promoting therapies, as activation of NPR-B subsequent to epiphyseal closure is not anticipated to contribute to increases in longitudinal height.

Future drug design will probably be guided less by whether a molecule is a CNP analog and more by how exposure is controlled. In this framework, longer systemic exposure is not intrinsically superior; it is advantageous only when it matches the biological window, tissue compartment, reversibility requirement, and safety profile of the intended indication. Daily vosoritide, once-weekly navepegritide, long-acting BMN 333, depot-compatible [Gln6,14]CNP-38, collagen-binding CNP-37, Fc-fusion NC-2, and CNP-ELP fusions represent different solutions to the same problem: native CNP biology is effective but too short-lived for convenient therapy (Breinholt et al., 2019; Hirai et al., 2025; James and James, 2017; Jowett et al., 2021; Lorget et al., 2012; MacEwan and Chilkoti, 2014; Ono et al., 2013; Schneider et al., 2022; Wendt et al., 2015; Author Anonymous, 2026c). The ideal design may differ by indication. For pediatric systemic therapy, smoother and longer exposure may reduce injection burden and improve adherence. For craniofacial or skull-base disease, early and sufficient exposure during the relevant growth window may be particularly important. For osteoarthritis or fracture repair, local matrix retention may be preferable to broad systemic distribution. For rare severe disorders, one-time or infrequent ligand-delivery strategies may be attractive, but the reversibility and safety of sustained pathway activation must be addressed.

A tailored and individualized approach will be essential as NPR-B agonist therapy extends to various skeletal-growth disorders. The genotype alone may not reliably predict treatment response, particularly in cases of partial *NPR2* deficiency, RASopathies, or *ACAN*-related short stature (Badawi et al., 2023; Choi et al., 2012; Dauber et al., 2026; Inoue et al., 2019; Jeong et al., 2026; Ke et al., 2021; Muthuvel et al., 2024; Ono et al., 2013; Steen et al., 2017). Functional assays assessing CNP-stimulated cGMP production, biomarkers such as NT-proCNP or urinary cGMP, and imaging markers of growth-plate or cartilage activity may aid in identifying responders and optimizing dosing strategies (Chan et al., 2022; Chen et al., 2023; Dauber et al., 2026; Espiner et al., 2019; Irfanullah et al., 2018; Jeong et al., 2026; Wei et al., 2025). Nevertheless, biomarkers should be interpreted with caution. While a pharmacodynamic signal confirms pathway engagement, it does not necessarily indicate improvements in final height, skeletal proportion, joint structure, or overall quality of life (Dauber et al., 2026; Galetaki et al., 2026). Therefore, future clinical trials should incorporate molecular, biochemical, radiographic, and clinical endpoints in combination, rather than depending solely on a single marker.

Long-term safety will remain a central concern. NPR-B agonists influence a pathway crucial to skeletal growth, vascular tone, and tissue remodeling. Established short-term treatment-related side effects differ by agent and formulation but include injection-site reactions, gastrointestinal symptoms, pain in an extremity, immune reactions, and transient decreases in blood pressure. In contrast, disproportionate or accelerated growth, genu valgum, slipped capital femoral epiphysis, scoliosis, changes in bone mineral density, and effects on craniofacial, spinal, or reproductive development are potential long-term orthopedic or developmental concerns requiring surveillance, not established causal adverse reactions. These categories should be reported separately as treatment begins earlier, lasts longer, or expands to new indications (Breinholt et al., 2019; Chan et al., 2022; Dauber et al., 2026; Savarirayan et al., 2019; Savarirayan et al., 2020; Author Anonymous, 2026c). Safety considerations may become increasingly complex if treatment commences in infancy, is combined with human growth hormone or disease-specific therapies, or is applied in conditions where the primary defect does not directly involve the FGFR3-MAPK or CNP/NPR-B axis.

Ultimately, a more comprehensive understanding of the structural and mechanistic aspects of human NPR-B is anticipated to facilitate the development of structure-guided agonists. Currently, the lack of an experimentally resolved structure of human NPR-B constrains precise analysis of ligand binding, receptor activation, and pathogenic variants of *NPR2*. High-resolution structures of human NPR-B in both inactive and CNP-bound active conformations would underpin the rational design of receptor-specific peptides, tissue-specific agonists, biased or partial agonists, and potentially non-peptide modulators. Such advancements would be particularly advantageous if the field progresses from general NPR-B activation to the customization of the intensity, duration, and tissue-specific localization of signaling pathways, tailored to individual disease contexts.

Overall, the future of this strategy is likely to be defined by two parallel objectives. The first objective is to establish NPR-B agonism as a disease-modifying treatment for well-matched pediatric growth-plate disorders, with outcomes that extend beyond growth velocity. The second objective is to adapt CNP/NPR-B biology for localized repair of cartilage and bone, in which controlled tissue retention may be more important than systemic exposure. Progress in both areas will depend on aligning the molecular design of each agent with the biological requirements of the target disease.

## 6 Conclusion

The CNP/NPR-B/cGMP pathway has advanced from a physiological regulator of growth-plate cartilage to a clinically actionable therapeutic target for skeletal diseases. Foundational research established its involvement in chondrocyte proliferation, hypertrophic differentiation, extracellular matrix synthesis, and endochondral ossification. Human genetic studies have revealed that either insufficient or excessive NPR-B signaling correlates with contrasting skeletal growth phenotypes. The subsequent development of CNP-derived agonists has demonstrated that activating this pathway can be translated into effective treatments, particularly evident in FGFR3-related achondroplasia.

The agents analyzed herein demonstrate that NPR-B agonism is not a singular, uniform strategy. Native CNP-22 and CNP-53, vosoritide, navepegritide, BMN 333, ASB20123, microsphere [Gln6,14]CNP-38 conjugate, collagen-binding CNP-37, NC-2, and CNP-ELP fusions exhibit significant variations in structure, stability, dosing intervals, and tissue distribution. These structural differences bear substantial biological significance. A systemically administered, long-acting agonist may be appropriate for pediatric growth-plate disorders, whereas a matrix-retained or locally delivered molecule may be more suitable for applications such as fracture repair, osteoarthritis, or cartilage regeneration. Similar structural changes can be interpreted meaningfully only when its pharmacokinetic profile, tissue exposure, pharmacodynamic pattern, dosing burden, intended use, and evidence tier are considered together.

Future progress will depend on matching each molecular design to the biological requirements of each target disorder. For achondroplasia and related pediatric growth disorders, important questions include not only increased annualized growth velocity but also final adult height, skeletal proportionality, skull-base and spinal complications, limb alignment, pain, physical function, and quality of life. For conditions beyond achondroplasia, including hypochondroplasia, partial *NPR2* deficiency, *NPPC*/CNP deficiency, specific RASopathies, *ACAN*-related short stature, acquired growth impairment, and local orthopedic diseases, the rationale and endpoints should be evaluated separately rather than assumed to be equivalent across indications.

Overall, NPR-B agonism illustrates how growth-plate biology can be converted into targeted skeletal medicine. Its potential extends beyond merely promoting longitudinal bone growth, to increasingly precise modulation of peptide stability, exposure, and tissue localization. Nonetheless, the broader clinical application necessitates meticulous long-term safety evaluations, validated biomarkers, comprehensive structural understanding of human NPR-B, and outcome metrics that extend beyond biochemical pathway activation. The principal challenge for the field is therefore to employ CNP/NPR-B biology with sufficient accuracy to enhance clinically meaningful skeletal outcomes while maintaining the equilibrium of growth-plate and tissue-remodeling physiology. Claims based primarily on sponsor disclosures, patents, or conference abstracts should remain provisional until supported by peer-reviewed pharmacology and clinical outcomes.
